# A Protective Vaccine against Chlamydia Genital Infection Using Vault Nanoparticles without an Added Adjuvant

**DOI:** 10.3390/vaccines5010003

**Published:** 2017-01-19

**Authors:** Janina Jiang, Guangchao Liu, Valerie A. Kickhoefer, Leonard H. Rome, Lin-Xi Li, Stephen J. McSorley, Kathleen A. Kelly

**Affiliations:** 1 Department of Pathology and Laboratory Medicine, David Geffen School of Medicine at UCLA, 10833 Le Conte Ave. CHS 1P-177, Los Angeles, CA 90095, USA; guangchaoliu@gmail.com (G.L.); kelly@mednet.ucla.edu (K.A.K.); 2 Department of Biological Chemistry, David Geffen School of Medicine at UCLA, Los Angeles, CA 90095, USA; vkick@ucla.edu (V.A.K.); LRome@mednet.ucla.edu (L.H.R.); 3 Center for Comparative Medicine, Department of Anatomy, Physiology & Cell Biology, School of Veterinary Medicine, University of California-Davis, Davis, CA 95616, USA; lxli@ucdavis.edu (L.-X.L.); sjmcsorley@ucdavis.edu (S.J.M.)

**Keywords:** *Chlamydia*, infectious diseases, vaults, mucosal immunology, CD4, tetramer

## Abstract

*Chlamydia trachomatis* genital infection is the most common sexually transmitted bacterial disease, causing a significant burden to females due to reproductive dysfunction. Intensive screening and antibiotic treatment are unable to completely prevent female reproductive dysfunction, thus, efforts have become focused on developing a vaccine. A major impediment is identifying a safe and effective adjuvant which induces cluster of differentiation 4 (CD4) cells with attributes capable of halting genital infection and inflammation. Previously, we described a natural nanocapsule called the vault which was engineered to contain major outer membrane protein (MOMP) and was an effective vaccine which significantly reduced early infection and favored development of a cellular immune response in a mouse model. In the current study, we used another chlamydial antigen, a polymorphic membrane protein G-1 (PmpG) peptide, to track antigen-specific cells and evaluate, in depth, the vault vaccine for its protective capacity in the absence of an added adjuvant. We found PmpG-vault immunized mice significantly reduced the genital bacterial burden and histopathologic parameters of inflammation following a *C. muridarum* challenge. Immunization boosted antigen-specific CD4 cells with a multiple cytokine secretion pattern and reduced the number of inflammatory cells in the genital tract making the vault vaccine platform safe and effective for chlamydial genital infection. We conclude that vaccination with a *Chlamydia*-vault vaccine boosts antigen-specific immunities that are effective at eradicating infection and preventing reproductive tract inflammation.

## 1. Introduction

*Chlamydia trachomatis* infection is the major cause of bacterial sexually transmitted infections (STIs) with major adverse effects on female reproductive tract health and function. The magnitude of reproductive dysfunction and infertility associated with sexually transmitted *C. trachomatis* infection is a significant health burden with over 1.5 million infections annually in the United States, however, a vaccine has not yet been developed [1,2]. Many antigens have been identified as vaccine candidates and studies have shown a combination of antigens is more effective than a single antigen [3,4]. However, a major hurdle remains the identification of an adjuvant that does not induce a strong inflammatory response yet enhances protection from vaccination. Previous research has shown that major histocompatibility complex (MHC) class-II restricted interferon gamma (IFN-γ) producing cluster of differentiation 4 (CD4^+^) T cells are required for protective immunity [5,6,7]. Using mass spectrometry to screen chlamydial peptides eluted from MHC-II peptides, PmpG (polymorphic membrane protein G-1) was identified as conferring strong immunogenicity upon immunization [8]. Here, we used a recombinant vault nanoparticle packaged with PmpG as a candidate antigen to evaluate the immune response without an added adjuvant.

Vault nanoparticles are hollow barrel shape ribonucleoprotein complexes found in most eukaryotic organisms [9]. Native vaults consist of multiple copies of three different proteins: the major vault protein (MVP), vault poly adenosine diphosphate (ADP) ribosyl polymerase (VPARP), and telomerase-associated protein 1 (TEP1) [10]. When MVP is expressed in cells lacking vaults, hollow vault shells are assembled from 78 copies of MVP forming empty vault particles structurally indistinguishable from native vaults [11]. An MVP interaction domain (INT) originally identified in the VPARP protein associates non-covalently with an MVP binding site and can be used to internally package candidate vaccine protein antigens. We have shown recombinant vaults can be used to deliver antigens, inducing both adaptive and protective immunity, and they demonstrate superior protection to the intravaginal challenge. [12]. Moreover, the vault can be engineered to deliver drugs and promote anti-tumor responses [12,13,14]. Eko, FO et al. has shown that a vaccine that contains multiple antigens provides better protection than immunization with a vaccine containing only a single antigen [15]. In the current study, we test the ability of a different chlamydial antigen (PmpG) to provide protection. We used PmpG-vaults in a vaccine regimen without added adjuvant. We took advantage of a PmpG-tetramer, to examine antigen-specific protective immunity in the murine model. Our study revealed that PmpG-vault immunizations induced strong antigen-specific cellular immune responses featured by PmpG-tetramer+ CD4 T cells, which also produce multiple cytokines. Nearly 30% of tetramer+ CD4 T cells express central memory markers. Furthermore, PmpG-vault vaccinations generate strong protection and alleviate inflammation in the genital tract as defined by fewer neutrophils and tumor necrosis factor alpha (TNF-α) secreting cluster of differentiation 8 (CD8) T cells and reduced genital tract pathology. In summary, we demonstrated that recombinant vaults can be used to deliver antigens, inducing both adaptive and protective immunity.

## 2. Material and Methods

### 2.1. Mice

female C57 BL mice, 6–8 weeks old, were purchased from Charles River Laboratory (San Diego, CA, USA). Animals were housed according to the American Association of Laboratory Animal Care guidelines. All animal experimental procedures were approved by the University of California at Los Angeles (UCLA) Institutional Animal Care and Use Committee and were conducted according to relevant national and international guidelines. Procedures were designed to provide for maximum comfort and minimal stress to the animals. The animals were monitored during experiments for signs of agitation (licking, biting, or guarding the vaginal region), failure to groom, loss of appetite, or marked weight loss (>10%).

### 2.2. Expression and Purification of Recombinant Vaults

Vault vaccines were produced as previously described [12,16], PmpG (ASPIYVDPAAAGGQPPA) and the 385 amino acid coding region of ovalbumin were independently fused to the major vault protein interaction domain (INT). Quantitation of PmpG and Ovalbumin (OVA) after packaging into vaults was performed using NuPAGE 4%–12% precast gels. Gels were stained with MicroWave Blue Coomassie stain, scanned with an Epson high-resolution scanner and analyzed with ImageQuant 5.2 software (Nonlinear Dynamics Ltd., Newcastle, UK). 

### 2.3. Chlamydia Preparation, Immunization and Challenge of Mice

*Chlamydia muridarum* (MoPn) was grown on confluent McCoy cell monolayers, purified on Renograffin gradients, and stored at −80 °C in SPG buffer (sucrose-phosphate-glutamine) as previously described [17]. Mice receiving vaults or PBS were anaesthetized with a mixture of 10% ketamine plus 10% xylazine and immunized intranasally (i.n.) with 100 µg PmpG- or OVA-vaults in 10 uL saline for a total of three times intranasally at two week intervals. Previously, we have checked mouse lung histology at different time points post intranasal inoculum. We observed slight infiltration and hypercellularity in lung histology at early time point post treatment, but this infiltration soon dissolved. Mice were hormonally synchronized by subcutaneous injections with 2.5 mg of medroxyprogesterone acetate (Depo Provera, Upjohn, Kalamazoo, MI, USA) in 100 μL saline 7 days prior to a vaginal challenge with 1.5 × 10^5^ infusion forming units (IFUs) of *C. muridarum* under anesthetization. Infection was monitored by measuring inclusion forming units from cervical-vaginal swabs (Dacroswab Type 1, Spectrum Labs, Rancho Dominguez, CA, USA).

### 2.4. Histology, Hematoxylin, and Eosin and Trichrome Stain

Genital tracts (GTs) were removed and the oviducts were assessed for hydrosalpinx: 1+ = just barely visible; 2+ = 2.5 mm diameter; 3+ = 3.5 mm diameter; and 4+ ≥ 4.5 mm diameter. The GT tissues, were removed at day 49 post-infection or 60 days after the last immunization, fixed in 10% formalin overnight, followed by 70% ethanol. Tissues were embedded in paraffin blocks, sectioned (5 µm), and then stained with hematoxylin and eosin. Four oviduct sections per mouse, obtained near the ovary, were stained with hematoxylin and eosin (H&E), masked for identity, and scored for acute inflammatory cells (neutrophils); 0 = normal; 1 = rare foci; 2 = scattered (1 to 4) aggregates; 3 = numerous aggregates (>4); and 4 = severe diffuse infiltration [18,19]. A second set of four oviducts per mouse were stained with hematoxylin and eosin followed by Gormori trichrome [20]. Trichrome stained slides were used to assess fibrosis and were masked and counted using the following scoring scheme: 1 = bright blue staining collagen fibers surrounding <33% of oviducts; 2 = bright blue staining collagen fibers surrounding 34%–66% of oviducts; 3 = bright blue staining collagen fibers surrounding >66% of oviducts. An Aperio Imagescope was programed to measure the area of the oviduct lumen (Oviduct dilation) and the tissue between the oviduct wall and epithelial cell layer (Oviduct submucosa) by outlining the lumen or submucosal tissue according to the product manual (Leica Microsystems Inc., Buffalo Grove, IL, USA). Two slides were scored and averaged per oviduct.

### 2.5. Gel Electrophoresis and Immunoblotting

Sodium dodecyl sulfate-polyacrylamide gel electrophoresis was performed using the discontinuous buffer system and 10%–12.5% acrylamide gels as previously described [12]. Vaults were transferred to an Immunbiolon-P transfer membrane (Millipore, Billerica, MA, USA) and blocked with 5% (wt/vol) nonfat dry milk in PBS-0.1% Tween 20. Membranes were individually incubated for 1 h with sera from immunized mice (1:500) or rabbit anti-MVP (1:500, MAB 2013, Santa Cruz Biotechnology Inc., Santa Cruz, CA, USA) followed by a 1 h incubation with horseradish conjugate (1:5000, Amersham Biosciences, Piscataway, NJ, USA). Bound conjugates were detected with SuperSignal West Dura extended duration substrate (Pierce Biotechnology Inc., Thermo Scientific, Rockford, IL, USA) and an Alpha Innotech Flurochem 8000 imager.

### 2.6. Isolation of Lymphocytes and Tetramer Enrichment

Lymphocytes were harvested from four tissues of individual mice: Spleen (Spl); iliac lymph nodes (iLN), the local draining lymph nodes for the reproductive tract; middle portion of the genital tract (MGT) or uterine horn which is the area from the end of the oviduct tissue to the bicornuate uterus tissue; and oviducts, by dissociating the tissues into single cell suspensions as described below. Briefly, the MGT or oviducts were removed and cut into 0.5 cm pieces that were then rinsed with Ca^2+^Mg^2+^-free Hanks’ balanced salt solution (HBSS). The tissue was incubated in a mixture of 5 mM ethylenediaminetetraacetic acid (EDTA) in HBSS at 37 °C for two 15 min periods with gentle stirring. The tissue was then incubated with RPMI 1640 containing 10% bovine calf serum, antibiotics, 25 mM HEPES and 1.5 mg/mL collagenase (Sigma, St. Louis, MO, USA) and incubated at 37 °C with stirring for two—1 h periods. The MGT or oviduct isolated cells were separated on a 40% over 75% discontinuous Percoll gradient (Pharmacia, Piscataway, NJ, USA) and centrifuged at 2000 rpm at 22 °C for 20 min. Mononuclear cell pellets were resuspended in RPMI 1640 at 4 °C until used [21,22]. Spl and iLN tissue were minced using 70 µm cell drainer (BD Falcon), individual cells were washed with 1% PBS followed by red blood cell lysis treatment. Lymphocytes were resuspended in RPMI 1640 stored at 4 °C until used. 

### 2.7. Tetramer Enrichment and Flow Cytometry Staining

Single cells suspensions in FACS buffer (PBS with 1% BSA) were stained with Phycoerythrin (PE)-PmpG-tetramer for 1 h in the dark as described in [23]. Cells were washed and tetramer positive cells were enriched using an EasySep PE Positive selection kit (STEMCELL Technologies, Vancouver, BC, Canada). Enriched cells were later used for further analysis.

For intracellular cytokine staining, lymphocytes isolated from different organs were incubated in RPMI 1640 in the presence of PmpG_303-311_ peptide for 6 h. Brefeldin A (Sigma) was added 4 h before the end of culture. To exclude dead cells, samples were stained using Live/Dead fixable dead cell stain kits (Life Technologies, Grand Island, NY, USA), and following surface staining, cells were directly stained with various FACS antibodies: fluorochrome-labeled antibodies against CD3 (clone 145-2C11), CD4 (clone GK1.5), CD8 (clone 53-6.7), CD44 (clone IM7), CD62L (clone MEL-14), Gr-1 (clone RB6-8C5), NK1.1, (clone PK136), CD11c (clone N418), CD19 (clone 6D5), CD11b (clone M1/70), CD40 (clone 3/23), CD69 (clone H1.2F3) (Biolegend, San Diego, CA, USA) and PmpG-tetramer for 20 min on ice [19]. After being washed, the cells were incubated with Cytofix/Cytoperm (BD Biosciences, San Jose, CA USA ) for 1 h and then stained with fluorochrome-conjugated anti-IFN-γ (clone XMG1.2), IL-2 (clone ES5-16E3), TNFα (clone MP6-XT22) or interleukin (IL)-17 (clone TC11-18H10.1) antibody (Biolegend) for 30 min on ice, washed again, resuspended in staining buffer with 1% PFA and analyzed on BD LSR II (Beckman Dickinson, Franklin Lakes, NJ, USA). FACS data was analyzed by Flowjo (Tree Star, Ashland, OR, USA).

### 2.8. Statistical Analysis

Statistical significance was determined between two groups using a two tailed *t*-test. Statistical differences among three or more groups were assessed using two-way repeated measured analysis of variance (ANOVA) followed by Tukey’s post-hoc test for comparing the chlamydial course of infection following MoPN infection. Kruskal-Wallis and Dunn’s multiple comparison post-hoc tests were used to compare histologic scores. ANOVA followed by Bonferroni post-hoc tests were used to compare flow cytometry data [17]. GraphPad Prism version 5.04 was the statistical software used (GraphPad Software, San Diego, CA, USA). Groups were considered statistically different at *p* < 0.05. Data are presented as mean ± standard deviation (SD) or standard error of the mean (SEM) as indicated.

## 3. Results

### 3.1. PmpG-Vault Immunization Reduces Early Bacterial Burden Following Chlamydial Genital Infection and Does Not Induce Systemic Inflammation

**Figure 1:**
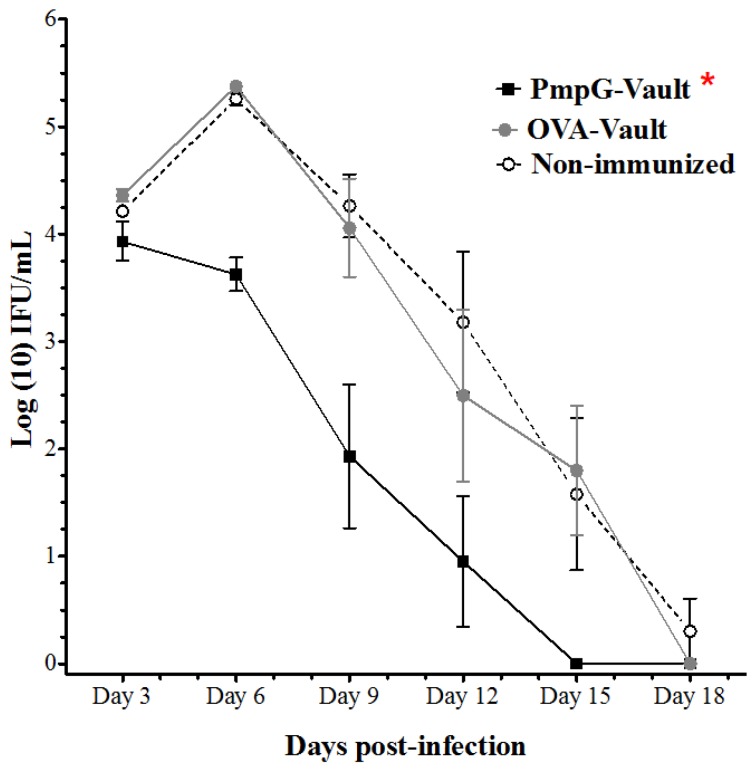
Polymorphic membrane protein G-1 (PmpG)-vault immunization decreases bacterial burden following intravaginal challenge. The bacterial burden of chlamydiae in the genital tract was assessed in mice that were intranasally (i.n.) immunized with PBS, PmpG-vaults, or OVA-vaults followed by *Chlamydia muridarum* (MoPn) vaginal challenge as described in the materials and methods section. Vaginal swabs samples were collected throughout the course of MoPn infection and infusion forming units (IFUs) were determined and plotted as the group mean ± standard error of the mean (SEM). The IFU data was analyzed by two-way repeated measures (RM) ANOVA, *p* < 0.01, *n* = 6 mice/group. We have done experiments comparing PmpG-vault, mitochondrial outer membrane permiabilization (MOMP)-vault and live *Chlamydial muridarum* (Cm) immunization. PmpG-vault generated comparable protection against bacterial vaginal challenge to MOMP-vault and live Cm immunization.

To evaluate the level of protection conferred by PmpG-vault immunization, mice were immunized by the intranasal route with PmpG-vaults three times at 2 week intervals. Two controls were used; a specificity control with OVA-vaults, and an immunization control with PBS alone or a non-immunized group. No adjuvants were included with any of the immunizations. Mice were challenged with a dose of 1.5 × 10^5^ IFUs of *C. muridarum*, 2 weeks following the last immunization. The effect of immunization was first evaluated by monitoring the bacterial burden in vaginal swabs collected every 3 days as reported [17]. The PmpG-vault group showed significantly lower bacterial burden compared to the non-immunized or OVA-vault groups throughout the course of infection (Figure 1). More importantly, the PmpG-vault immunized group started with a comparable bacterial level as the other two groups, but the bacterial shedding increased by day 6 for the two control groups, while it was reduced by over a log, in the PmpG-vault immunized group. This trend continued and the PmpG-vault immunized group cleared infection 3 days earlier than the control groups.

**Figure A1:**
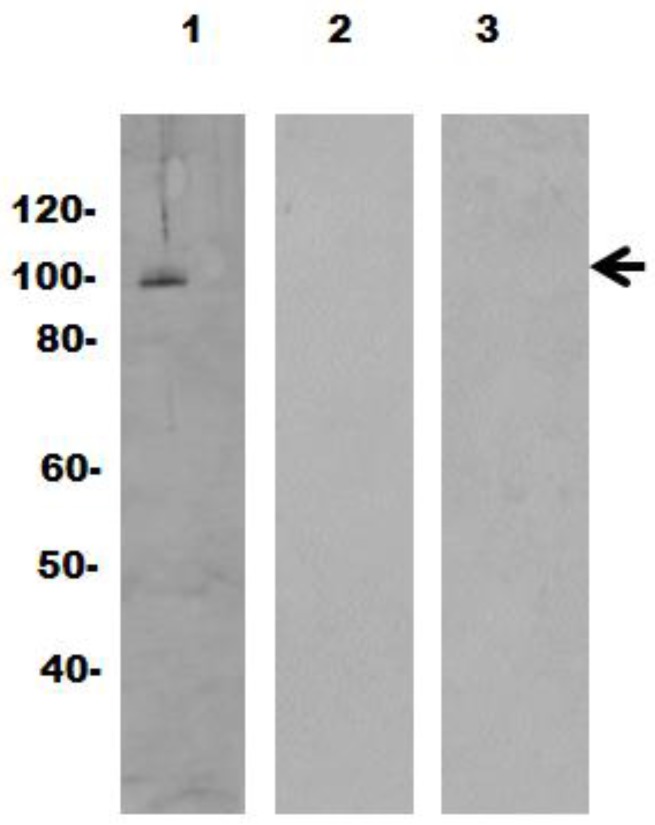
Immunization with vault vaccines do not induce antibody to the major vault protein (MVP). Western blot analysis of high speed pellet extracts of cp-MVP vaults [48] (1 ng/lane) probed with (**1**) rabbit anti-MVP antisera, (**2**) sera pooled from mice immunized with PmpG-vaults and (**3**) sera pooled from mice immunized with OVA-vaults. The sera were collected 7 weeks after immunization from mice shown in Figure 1. Molecular weight sizes are indicated. MVP (~97 kDa) is indicated (arrow head).

The engineered vault used to carry the PmpG antigen is entirely composed of a highly conserved protein, MVP, that exists in all mammalian cells. An immune response against MVP would not be expected. Previously, we found that vaults do not initiate production of inflammatory cytokines upon in vitro delivery to antigen presenting cells [12]. To determine if vaults themselves induce inflammation in vivo, we evaluated immunized mice for evidence of chronic inflammation. At 60 days following immunization with vaults containing a chlamydial protein (PmpG; *n* = 3 mice), the mice were necropsied and the major organs (liver, spleen, pancreas, kidney, thymus, heart, and brain) were histologically examined by the UCLA veterinary staff for evidence of chronic inflammation. Control groups included mice immunized with empty vaults (*n* = 3) and naïve mice (*n* = 3). In all cases, there was no chronic inflammation or fibrosis, evidence of sustained inflammation. Further investigation of sera pooled from the mice that were immunized with PmpG-vaults (Appendix A, Figure A1, lane 2) or OVA-vaults (Appendix A, Figure A1, lane 3) did not produce antibodies against the vault protein (MVP) as detected by Western blot analysis of empty vaults at a concentration that is naturally found in cells. The positive control (lane 1) identifies the MVP as shown by the arrowhead (Appendix A, Figure A1). Previously, we have checked mouse lung histology at different time points post intranasal inoculum . We observed slight infiltration and hypercellularity in lung histology at early time points post intranasal inoculation, but this infiltration soon dissolved. Taken together, intranasal delivery of a vault-vaccine carrying the PmpG peptide induced a strong protective immunity in the genital tract against a *C. muridarum* vaginal challenge. Vaults were a safe antigen delivery vehicle that did not induce chronic inflammation in vivo or an immune response against the vault protein. 

### 3.2. Genital Tract Histopathology Following C. muridarum Challenge is Reduced by Vaccination with Vaults Containing PmpG

**Figure 2:**
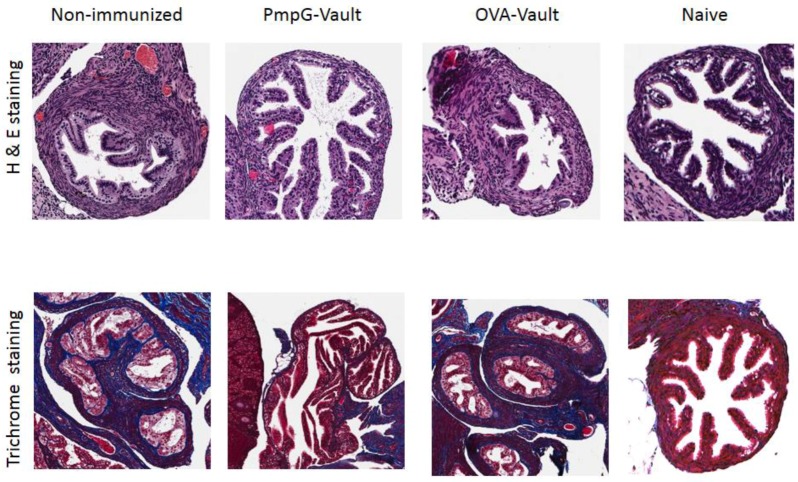
PmpG-vault vaccination prevents oviduct pathology. Representative photomicrographs of Hematoxylin, Eosin and Trichrome stained sections are shown from mice that were i.n. immunized with PBS (Non-immunized), PmpG-vaults, or OVA-vaults followed by a MoPn vaginal challenge as described in the materials and methods section. A naïve mouse is also shown for comparison. Oviduct tissues were harvested 49 days after vaginal infection, processed en bloc for paraffin sections and stained with H&E or Trichrome dye preparations as described in the materials and methods. Sections are at 20×.

**Figure 3:**
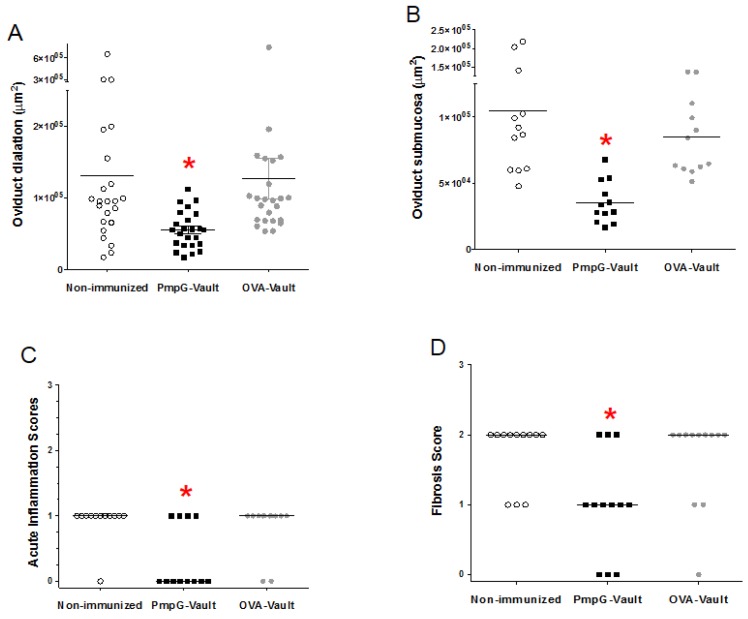
PmpG-vault vaccination reduces various measures of oviduct inflammation. Mice were i.n. immunized with PmpG-vaults, OVA-vaults, or PBS (Non-immunized) followed by a MoPn vaginal challenge as described in the materials and methods section. Oviduct tissue was harvested 49 days after vaginal infection and processed en bloc for paraffin sections. Slides were stained with H&E or trichrome and used for assessment of oviduct inflammation. Scatter plot and median (horizontal line) of: (**A**) Oviduct dilation from H&E stained slides, *n* = 12 mice or 24 oviducts/group; (**B**) Oviduct submucosal tissue thickness and median (horizontal line) measurements from H&E slides, *n* = 6 mice or 12 oviducts/group; (**C**) Acute inflammatory scores of oviducts as measured from H&E stained slides, *n* = 6 mice or 12 oviducts/group; (**D**) Fibrosis scores determined from trichrome slides of oviduct tissue, *n* = 6 mice or 12 oviducts/group. * *p* < 0.05, Kruskal-Wallis with Dunn’s Multiple Comparison post-hoc test.

Our previous studies using MOMP-vaults to immunize mice with this same i.n. immunization protocol, prevented histologic evidence of oviduct pathology following immunization. In this study, gross observation of GT tissues harvested from mice (Figure 1) 49 days after genital infection with *C. muridarum* can be seen in Figure 2. Histopathological examination of H&E and trichrome staining showed that the PmpG-vault immunized group visually had fewer signs of oviduct inflammation as compared to that of the non-immunized or OVA-immunized groups. Moreover, the oviducts from H&E or trichrome stained sections from PmpG-vault immunized mice was similar to the naïve group. Evaluation of the groups found that hydrosalpinx scores were significantly (*p* < 0.05) reduced in PmpG-vault immunized mice (non-immunized; median = 4.0, 95% confidence interval (CI) = 2.8–4.5; PmpG-vault immunized mice: median = 1.5, 95% CI = 0.1–2.9; and OVA-vault immunized mice; median = 3.5, 95% CI = 2.5–4.2; *n* = 6 mice/group). We also evaluated upper tract inflammation in more depth by measuring H&E and trichrome stained slides using the Aperio Image system as described in the materials and methods section. We found that mice immunized with PmpG-vaults had significantly less oviduct dilation and a thinner submucosa layer compared with that of the non-immunized and OVA-immunized groups as shown in Figure 3A,B. The PmpG-vault immunized group also had significantly lower numbers of acute inflammatory cells as shown by the lower acute inflammation score and a lower fibrosis score as compared to those of the non-immunized and OVA-vaults immunized groups (Figure 3C,D). In summary, this study finds that vaults are an effective delivery vehicle for PmpG and shows that vaccination with a PmpG-vault-vaccine reduces the bacterial burden of a chlamydial infection and reduces GT tissue pathology in mice following a genital challenge with *C. muridarum.*

### 3.3. Vault-Vaccines Induce Antigen-Specific IFN-γ Secreting CD4 Cells That Also Produce Multiple Other Cytokines and Contain Central Memory Cells

**Figure 4:**
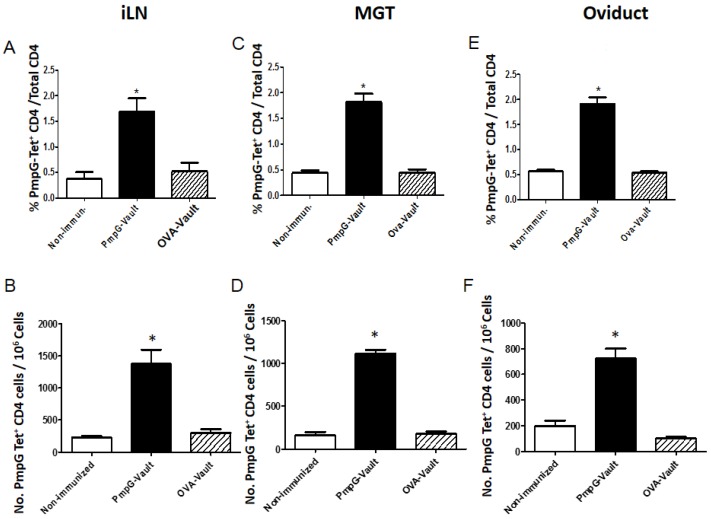
Antigen-specific T cell responses are increased by PmpG-vault vaccination. Mice were i.n. immunized with PBS (Non-immunized), PmpG-vaults, or OVA-vaults followed by a MoPn vaginal challenge as described in the materials and methods section. The iliac lymph nodes (iLN) (**A**,**B**), middle genital tract (**C**,**D**) and oviducts (**E**,**F**) were harvested from mice 7 days after genital infection. Single cell suspensions were labeled with anti-cluster of differentiation (CD)3, CD4, CD44 and PmpG-tetramer. In all cases, a minimum of 10^6^ events was acquired on the flow cytometer. Each graph shows the percentage (**A**,**C**,**E**) or number (**B**,**D**,**F**) of PmpG-tetramer^+^ CD4 T cells as indicated above. Results are representative of three independent experiments and are expressed as mean ± standard deviation (SD), *n* = 4/grp. The results were significant at * *p* < 0.001 by ANOVA and Bonferroni post-test.

**Figure A2:**
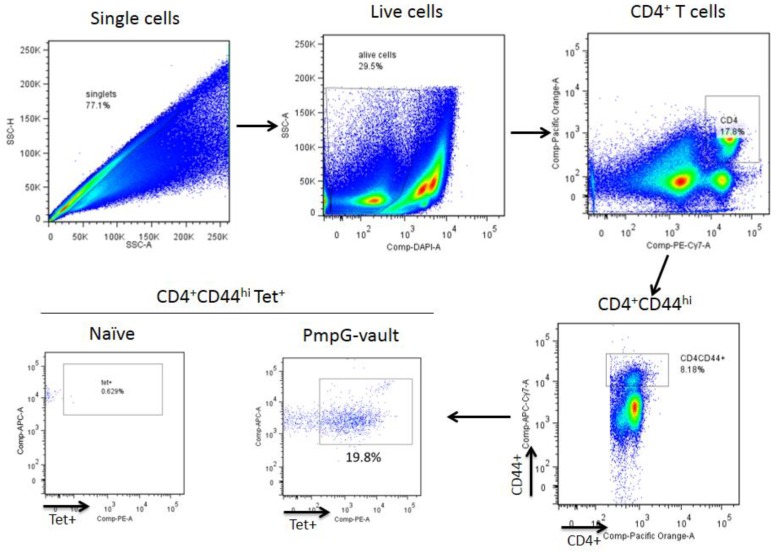
Gating scheme for flow cytometry analysis. Lymphocytes were isolated from the iLN and stained for the markers identified above and described in the materials and methods. The cells were first gated to exclude doublets or groups of cells, then live cells were selected by staining with a vital dye followed by selection of CD4^+^CD3^+^ cells. Cells staining positive for the PmpG-tetramer were identified in the population of CD4^+^CD44^hi^ cells. The cells could be further identified by cytokine production or expression of various cell surface markers.

Local antigen-specific cellular immune responses in the genital tract (GT) and draining lymph nodes were measured following the chlamydial challenge. Figure 4 shows a comparison of PmpG-tetramer CD4 cells in the iLN, MGT, and oviducts from mice that were i.n. immunized with PBS (Non-immunized), PmpG-vaults, or OVA-vaults followed by a MoPn vaginal challenge as described in the materials and methods section. Lymphocytes were isolated 7 days after infection and then stained for various markers. We focused on only CD4^+^ T cells that stained positive for the PmpG-tetramer (PmpG-Tet^+^). Single cells were identified by side scatter analysis of height and area and live lymphocytes were selected by staining with a vital dye as described in the materials and methods section. These cells were further partitioned into CD3^+^CD4^+^ cells that expressed CD44. This identification scheme is shown in Figure A2. We found that only PmpG-vault immunization significantly increased the percentage and number of antigen-specific PmpG-Tet^+^ CD4^+^ T cells in comparison to non-immunized (PBS immunized followed by MoPn infection) and OVA-vault immunized groups (Figure 4). The level of PmpG-Tet^+^ CD4^+^ T cells were significantly increased in the iLN, MGT, and oviduct of PmpG-vault immunized mice, regardless if PmpG-tetramer cells were determined by percentage of total CD4^+^ T cells (A,C,E) or absolute numbers (B,D,F).

**Figure 5:**
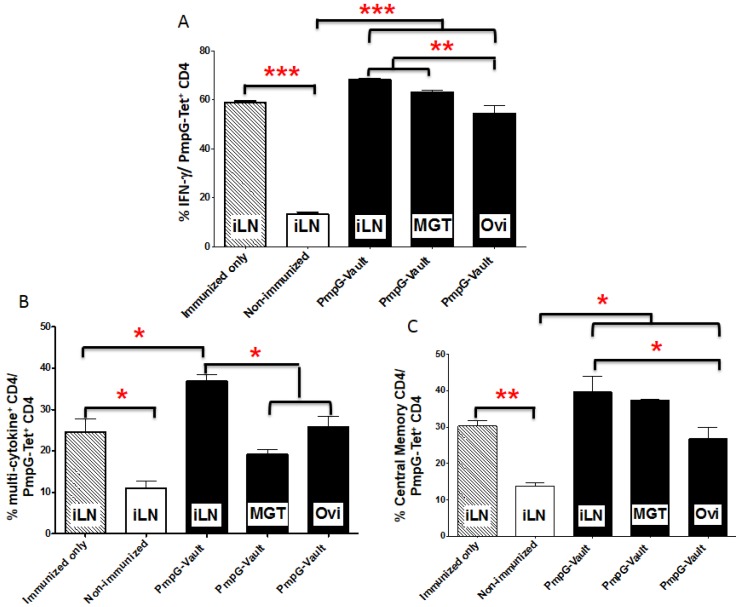
Cytokine production and memory cell markers on antigen-specific T cells following PmpG-vault vaccine immunization. Mice were i.n. immunized with PmpG-vaults (Immunized only), PBS (Non-immunized), or PmpG-vaults. All groups except the “Immunized only” group were given a MoPn vaginal challenge as described in the materials and methods section. Lymphocytes were isolated from various locations (iLN, middle genital tract (MGT), oviduct) 7 days after a challenge vaginal infection. The harvested lymphocytes were enriched for PmpG-tetramer (Tet)+CD4 T cells by magnetic columns and stained for intracellular cytokine production staining by FACS. (**A**) The percentages of interferon gamma (IFN-γ) PmpG-Tet+ CD4+ T cells among PmpG-Tet+ CD4+ T cells was plotted as the mean ± SD; (**B**) The percentages of PmpG-Tet+ CD4+ T cells producing multiple cytokines: IFN-γ, tumor necrosis factor alpha (TNF-α) and interleukin (IL)-17 among PmpG-Tet+ CD4+ T cells was plotted as the mean ± SD; (**C**) The percentages of central memory T cells (CD62Lhi memory CD4T cells) were plotted among PmpG-Tet+ CD4+ T cells as the mean ± SD. Results are representative of three independent experiments (*N* = 4/5 mice) were significant at *** *p* < 0.0001; ** = *p* < 0.01; and * = *p* < 0.05 by ANOVA.

We next characterized the PmpG-Tet^+^ CD4^+^ T cells for cytokine production and expression of memory cell markers by first enriching for PmpG-Tet^+^ CD4^+^ T cells using magnetic columns. We then quantified the percentage of various cell types within the isolated PmpG-Tet^+^ CD4^+^ T cells using the gating scheme described in Figure A2 [23]. Mice were i.n. immunized with PmpG-vaults (Immunized only), PBS (Non-immunized) or PmpG-vaults. All groups except the “Immunized only” group were given a MoPn vaginal challenge as described in the materials and methods section. As shown in Figure 5A–C, immunization with the PmpG-vault vaccine resulted in a significant increase of CD4 PmpG-Tet^+^ CD4^+^ T cells that secreted IFNγ, multiple cytokines (IFN-γ, TNF-α, and IL-17), or central memory cells (CD62hi) in the iLN compared to mice that were not immunized with vaults (PBS immunized and infection with MoPn). Comparison of iLN and GT (MGT and Ovi) tissues from immunized and challenged mice revealed a similar pattern of PmpG-Tet^+^ CD4^+^ T cells that only secreted IFNγ or expressed central memory markers but not those that produced multiple cytokines (IFN-γ, TNF-α, and IL-17). The development of multiple cytokine producing cells in immunized mice are considered a marker of protection for this infection [24]. However, it is possible that the kinetics of T cell trafficking is accelerated in PmpG-vault-immunized mice compared to that of mice immunized with antigens mixed with conventional adjuvants. This data possibly means that the expression of TNF-α and/or IL-17 in GT tissues are negatively correlated with infection or these cells have trafficked back to the secondary lymphoid tissue, explaining the effective control of infection within the first few days of vaginal infection. Interestingly, the Ovi tissue showed fewer numbers of PmpG-Tet^+^ CD4^+^ T cells expressing IFNγ, multiple cytokines, and T central memory (Tcm) compared to MGT tissue. This supports the findings that immune responses differ with respect to regional differences within the GT [17,25,26].

We were particularly interested in whether immunization alone produced different PmpG-Tet^+^ CD4^+^ T cell types or amounts compared to immunized and infected mice. However, analysis was limited to the secondary lymphoid tissue in mice that were not given a vaginal infection since undetectable numbers of PmpG-Tet^+^ CD4^+^ T cells were found in the GT of these mice. As seen in Figure 5A–C, the pattern of IFN-γ secreting and central memory PmpG-Tet^+^ CD4^+^ T cells in the iLN were similarly increased in mice only immunized with PmpG-vaults but not vaginally infected compared to that of mice immunized and infected. However, multiple cytokine producing PmpG-Tet^+^ CD4^+^ T cells were significantly lower in mice that were only immunized and not infected. Thus, infection appears to be important for inducing PmpG-Tet^+^ CD4^+^ T cells that can produce multiple cytokines; IFN-γ, TNF-α, and IL-17 which may only marginally aid in protection from bacterial load in the GT and reproductive tract inflammation.

### 3.4. Induction of Systemic Antigen-Specific Cellular Immune Responses

**Figure 6:**
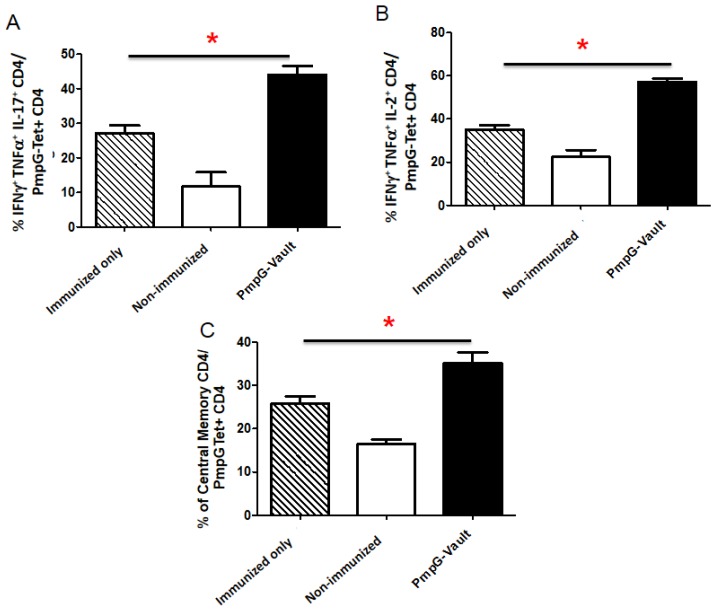
Intranasal mucosal immunization induces systemic immunity in spleen. Mice were i.n. immunized with PmpG-vaults (Immunized only), PBS (Non-immunized), or PmpG-vaults. All groups except the “Immunized only” group were given a MoPn vaginal challenge as described in the materials and methods section. Lymphocytes were isolated from the spleen on day 7 post challenge and enriched for PmpG-Tet+ CD4 cells. The percentage of cytokine or memory cells were determined. (**A**) The percentage of IFNγ^+^, TNFα^+^, IL-17^+^ cells within the PmpG-specific CD4 cell population in the spleen; (**B**) The percentage of PmpG-specific CD4 cells producing IFNγ^+^, TNFα^+^, IL-2^+^ in the spleen; (**C**) The percentage of central memory cells (CD62Lhi memory CD4T cells) within the PmpG-specific CD4 cell population in the spleen. Representative experiment includes three experiments. *n* = 4–5/group. * *p* < 0.05 (ANOVA).

In order to determine if delivery of vault-vaccines through the nasal mucosa also induce systemic as well as tissue specific cellular immunity, we examined the cellular immune responses in the spleen. Mice were i.n. immunized with PmpG-vaults (Immunized only), PBS (Non-immunized), or PmpG-vaults. All groups except the “Immunized only” group were given a MoPn vaginal challenge. CD4^+^ T cells were isolated from spleens 7 days after infection and enriched for PmpG-Tet^+^ CD4^+^ T cells using magnetic columns. The proportion of cytokine production and expression of memory cell markers within cells were quantitated using the gating scheme in Figure A2. [23]. Our results (Figure 6A,B) showed that the proportion of PmpG-Tet^+^ CD4^+^ T cells secreting multiple cytokines; (A) IFNγ^+^, TNFα^+^, IL-17^+^ or (B) IFNγ^+^, TNFα^+^, IL-2^+^ were significantly higher in the PmpG-vault immunized group compared to that of the Immunized only and Non-immunized groups. This pattern was also seen in central memory cells among PmpG-Tet^+^ CD4^+^ from PmpG-vault immunized only and immunized and infected groups (Figure 6C). This finding suggests that PmpG-vault immunization via a mucosal route is able to induce the formation of antigen-specific CD4^+^ cells with Tcm phenotype in a lymphoid tissue such as the iLN (Figure 5). Together, our data indicate that mucosal immunization induces cellular immunity in the mucosal and systemic circulation systems.

### 3.5. Inflammatory Factors Related to Tissue Pathology

**Figure 7:**
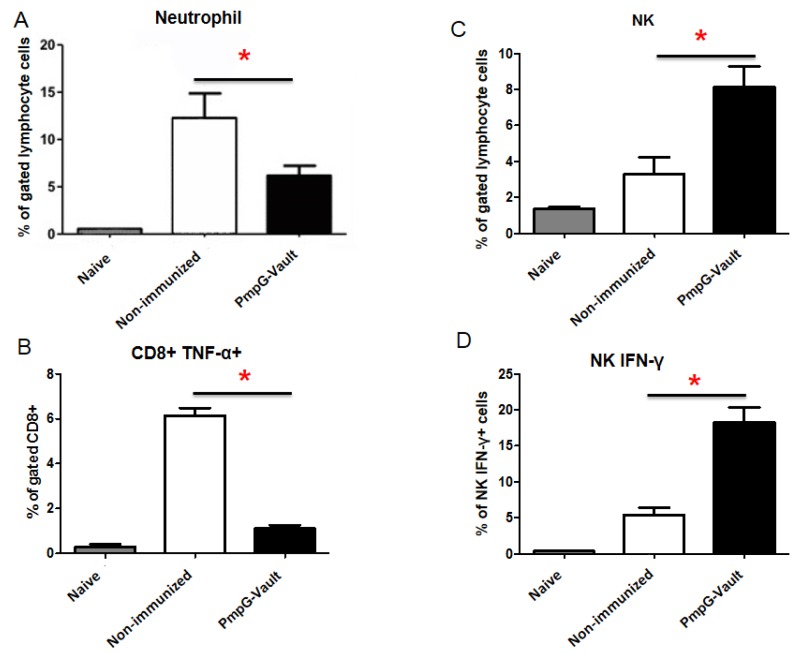
Immunization with a PmpG-vault vaccine reduces inflammatory responses in oviduct tissue. Mice were i.n. immunized with PBS (Non-immunized) or PmpG-vaults. All groups except the Naïve group were given an MoPn vaginal challenge as described in the materials and methods section. Frequency of (**A**) neutrophils; (**B**) CD8 TNF-α^+^ cells; (**C**) natural killer cell (NK); and (**D**) NK IFN-γ^+^ cells. Cells were harvested from oviducts 3 (**A**,**C**,**D**) or 7 (**B**) days following infection and processed for FACS analysis. Results are representative of three independent experiments (*n* = 4–5/group) and expressed as mean percent ± SD. The results were statistically significant at *p* < 0.05, with ANOVA and Bonferroni post-hoc test.

Pathologic consequences of chlamydial infection, including pelvic inflammatory disease (PID), infertility, and ectopic pregnancy, are a major public health concern and are associated with inflammatory responses that contribute to reproductive tissue dysfunction [27]. We wanted to determine if PmpG-vault vaccination would reduce inflammatory immune cells that have been associated with developing pathological consequences of chlamydial infection. In Figure 7 we examined the number of neutrophils, NK cells, IFN-γ secreting NK cells, and TNF-α secreting CD8 T cells within oviduct tissues of mice immunized with PmpG-vaults vaccination to determine if these inflammatory cell types were reduced compared to those that were only infected with MoPn, as these cell types are associated with oviduct inflammation [27,28]. Mice were i.n. treated with PBS (Non-immunized) or PmpG-vaults. All groups except the naïve group were given an MoPn vaginal challenge. We harvested the oviducts at differing times when the numbers of various immune cells were at their peak after infection and analyzed by flow cytometry. Our results indicated that mice immunized with PmpG-vaults had lower numbers of neutrophils and TNF-α secreting CD8 T cells compared to those of the non-immunized group. NK cells have been associated with increased clearance of *C. muridarum* from the genital tract [27,29,30]. Analysis of functional NK cells or those secreting IFN-γ, revealed that an increased number were found in the oviducts of mice immunized with the PmpG-vault vaccine shortly after infection. Taken together, our data show that vault vaccines not only induce antigen-specific cells but alter the quality of the immune response as we have reported for OVA-vault immunized mice [13]. Thus, vaults are more than just a delivery nanocapsules and appear to have specific adjuvant properties inducing a cellular immune response with unique attributes which is effective at reducing genital infection and also preventing genital tract inflammation.

## 4. Discussion

Studies from multiple labs have identified CD4 IFN-γ producing cells as the predominant cell type important for eradicating chlamydial genital infection. In this study, we characterized the antigen-specific CD4 cell immune response induced by an engineered vault-vaccine. PmpGs primarily contain T cell epitopes and would be expected to induce a T cell response [31]. We packed a peptide of a chlamydial outer membrane protein, PmpG, inside vault shells and used that preparation to immunize mice. Although this immunization regimen was expected to increase the number of PmpG-specific CD4 cells following immunization compared to that of mice that were only given a chlamydial genital infection, we were surprised that a similar percent of PmpG-specific CD4 T cells was found in iLN as compared to that in the GT. The 4 to 5-fold increase in PmpG-CD4 cells was sufficient to significantly reduce early infection in the GT. Others have found that delivery of a combination of antigens is more effective in protecting mice than a vaccine designed with a single chlamydial antigen [4,32]. Vaults are capable of holding multiple antigens and such a vault would possibly significantly lower or possibly prevent infection. In light of this proof of principle study, we are currently studying and optimizing a vault vaccine regimen for human use, using more advanced computerized technology to identify better antigen coverage of PmpG variants and other antigens (MOMP) in chlamydia trachomatis.

It is important that a vaccine designed to protect against chlamydial genital infection induces a “protective” immune response with little or no “inflammatory” response. Tracking PmpG-specific CD4 cells enabled us to compare the qualitative CD4 response among the group of mice that were immunized (PmpG-vault) and infected with MoPn, the group only immunized (Immunized only), and the group only infected (Non-immunized). Immunization prior to infection induced PmpG-specific CD4 cells in the iLN that produced significantly more IFN-γ only producing cells, multiple cytokine producing cells (IFN-γ, TNFα, and IL-17) [33] and a central memory subset. This differed from PmpG-specific CD4 T cells induced by a genital infection alone, and shows that immunization with vaults containing a protective chlamydial-specific protein induces protective cells. Tissue damage following a chlamydial genital infection is caused by the inflammatory response [34,35]. Our studies found that infection alone induced an inflammatory response characterized by a high level of inflammatory cells associated with genital tract inflammation (neutrophils and CD8, TNF-α producing cells) [27,28] compared to that of mice vaccinated with PmpG-vaults. These mice had significantly reduced numbers of neutrophils and CD8^+^ TNFα^+^ cells at levels seen in naïve mice. Consistent with other chlamydial vaccine studies, we noted a marked reduction in various histopathologic parameters and hydrosalpinx of GT tissues following PmpG-vault vaccination. The significant reduction of inflammatory cells by the PmpG-vault vaccine could explain the marked reduction in GT histopathology we observed in immunized mice following chlamydial genital infection. Our data demonstrates that vaccination of mice with protective chlamydial antigens in vaults induce “protective immune responses” and lessen the development of an inflammatory environment, suggesting that antigen-packaged vaults have unique adjuvant properties.

Examination of mucosal tissue provided additional information on the quality of the immune response following immunization. Interestingly, there was a significantly lower percent of IFN-γ producing and multiple cytokine secreting cells in the tissue, particularly in the oviduct or target tissue. In a previous study, we saw the number of IFN-γ producing cells increase following MOMP-vault vaccination in GT tissue by day 15 [12]. Possibly, the combination of PmpG with other chlamydial protective antigens or examination at a later time point would result in a higher percent of IFN-γ producing and multiple cytokine secreting CD4 T cells in GT tissues. Uniquely, our data show that vault-vaccination induces CD62Lhi central memory cells in spleen and iLN as expected but also in GT tissue. CD4 memory population is heterogeneous and does not follow the dogma that central memory is mostly found in lymph nodes and spleen [36,37,38]. We and others have found that CD4 T cells in GT tissue expresses inflammatory homing receptors (α4β1, E-selectin ligand) and inflammatory chemokine receptors (CXCL9, CXCL10) [17,25,39,40,41,42]. The inflammatory homing markers and chemokine receptors on CD4 memory cells specific for chlamydial antigens likely override CD62L and influence appearance in GT tissues. Following immunization, when the genital tract is re-exposed to chlamydiae antigens by a challenge infection, we postulate that Tcm in lymphoid organs migrate to genital tract tissues as Tcm has been shown to switch to T effector memory (Tem) phenotype and carry out effector function [43]. Following the clearance of infection, CD4 Tem modulate to Tcm and return to secondary lymphoid tissues. This migration pattern of Tcm was theorized based on our data of CD4 IFN-γ^+^ T cell migration to and from iLN during MoPn infection [12]. However, it is possible that Tcm remain in the local genital tract tissue for a time after immunization or infection since past literature has shown that mice are still protected from reinfection following immunization between 50–100 days after primary infection [44]. We only examined mice on day 7 after a genital infection, and future studies should be designed to evaluate this hypothesis and further the concept that a vault-vaccine regimen induces long-term memory immunity.

Vaccines designed from recombinant proteins have been shown to be safe but also have limited immunogenicity and require multiple injections and in some cases, booster injections to achieve and maintain sufficient protection from disease [45]. There are various approaches to designing a non-replicating vaccine. A vaccine that mimics the properties of pathogens has been successful [46]. Some examples include Inflexal (influenza virus, Crucell, Leiden, The Netherlands) and Engerix B (hepatitis B, GlaxoSimthKline, Brentford, UK). Interestingly, this approach has also resulted in successful vaccines against a vaginal pathogen such as Gardasil (HPV, Merck & Co., Kenilworth, NJ, USA) and Cervarix (HPV, GlaxoSmithKline). Vault-vaccines appear to mimic the *Chlamydia* development cycle by activating inflammasomes which have been shown to be necessary for enhancing lipid metabolism and supporting chlamydial growth [47]. However, unlike *Chlamydia*, vault-vaccines do not activate TLRs when administered in vivo and, therefore, do not induce excessive tissue inflammation, as we have shown previously [12] and in this study. In addition, vault-vaccines are on the same order of magnitude in size as an elementary body (EB). Vault-vaccines are approximately 0.1 µm whereas an EB is approximately 0.25 µm. Finally, vault-vaccines enter the endosomal pathway like *Chlamydia*. However, at least 50% of vaults appear in lysosomes effectively delivering the antigen for MHC II processing, whereas *Chlamydia* do not enter the lysosome and instead form an endosomal vacuole called the inclusion . The vault-vaccine mimics many properties of *Chlamydia* and this may be the major reason why vaults are effective in generating protection against chlamydial genital infection.
